# Clinical features and images of malignant lymphoma localized in the pancreatic head to differentiate from pancreatic ductal adenocarcinoma: a case series study

**DOI:** 10.1186/s12876-023-02779-3

**Published:** 2023-05-01

**Authors:** Naohiro Kato, Atsushi Yamaguchi, Syuhei Sugata, Takuro Hamada, Nao Furuya, Takeshi Mizumoto, Yuzuru Tamaru, Ryusaku Kusunoki, Toshio Kuwai, Hirotaka Kouno, Sho Tazuma, Takeshi Sudo, Miki Kido, Takuo Ito, Kazuya Kuraoka, Hiroshi Kohno

**Affiliations:** 1 Department of Gastroenterology, National Hospital Organization Kure Medical Center and Chugoku Cancer Center, Kure, Hiroshima Prefecture Japan; 2 Department of Surgery, National Hospital Organization Kure Medical Center and Chugoku Cancer Center, Kure, Hiroshima Prefecture Japan; 3 Department of Hematology, National Hospital Organization Kure Medical Center and Chugoku Cancer Center, Kure, Hiroshima Prefecture Japan; 4 Department of Pathology, National Hospital Organization Kure Medical Center and Chugoku Cancer Center, Hiroshima Prefecture Kure, Japan

**Keywords:** Malignant lymphoma, Pancreatic lymphoma, Pancreatic ductal adenocarcinoma, Pancreatic head, Adenocarcinoma

## Abstract

**Background:**

Pathological examination by endoscopic ultrasonography–guided fine-needle aspiration (EUS-FNA) has been reported to be useful in diagnosing pancreatic malignant lymphoma (ML), but some ML cases are difficult to be differentiated from pancreatic ductal adenocarcinoma (PDAC).

**Methods:**

This retrospective study included 8 patients diagnosed with ML that had a pancreatic-head lesion at initial diagnosis and 46 patients with resected PDAC in the pancreatic head between April 2006 and October 2021 at our institute. ML and PDAC were compared in terms of patients’ clinical features and imaging examinations.

**Results:**

The median tumor size was larger in ML than in PDAC (45.8 [24–64] vs. 23.9 [8–44] mm), but the median diameter of the caudal main pancreatic duct (MPD) was larger in PDAC (2.5 [1.0–3.5] vs. 7.1 [2.5–11.8] mm), both showing significant differences between these malignancies (both, *P* < 0.001). In the analysis of covariance, MLs showed a smaller caudal MPD per tumor size than PDACs, with a statistical difference (*P* = 0.042). Sensitivity and specificity using sIL-2R ≥ 658 U/mL plus CA19-9 < 37 U/mL for the differentiation of ML from PDAC were 80.0% and 95.6%, respectively.

**Conclusions:**

Diagnosing pancreatic ML using cytohistological examination through EUS-FNA can be difficult in some cases. Thus, ML should be suspected if a patient with a pancreatic tumor has a small MPD diameter per tumor size, high serum sIL-2R level, normal CA19-9 level. If the abovementioned features are present and still cannot be confirmed as PDAC, re-examination should be considered.

## Background

**Fig. 1:**
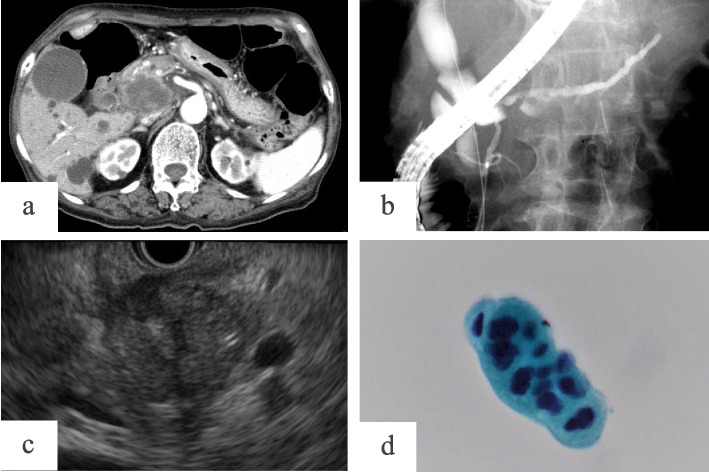
Case 1.1: A case of pancreatic lymphoma that was cytologically prediagnosed as a pancreatic ductal adenocarcinoma (PDAC). A 76-year-old woman was admitted to our hospital because of acute pancreatitis. Enhanced computed tomography (**a**) showed a 40 mm hypovascular tumor in the pancreatic head, and endoscopic retrograde pancreatography (**b**) displayed slight dilation of the caudal MPD. In cytologic assessment, we used tumor samples (**c**) obtained from endoscopic ultrasonography–guided fine-needle aspiration, (**d**) which then revealed atypical cells (papanicolaou stain, × 400) suggesting a PDAC, resulting in surgery

**Fig. 2:**
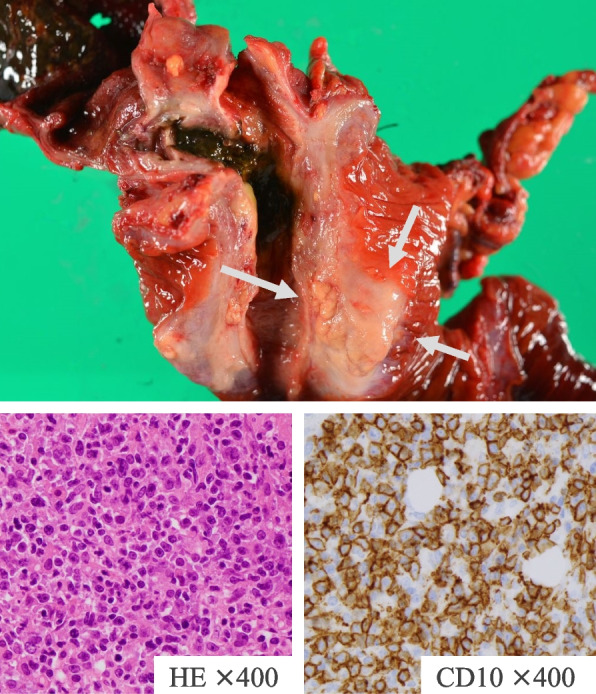
Case 1.2: Pathologically, the pancreatic head tumor shown in the final diagnosis was primary pancreatic diffuse large B-cell lymphoma in the pancreatic head

**Fig. 3:**
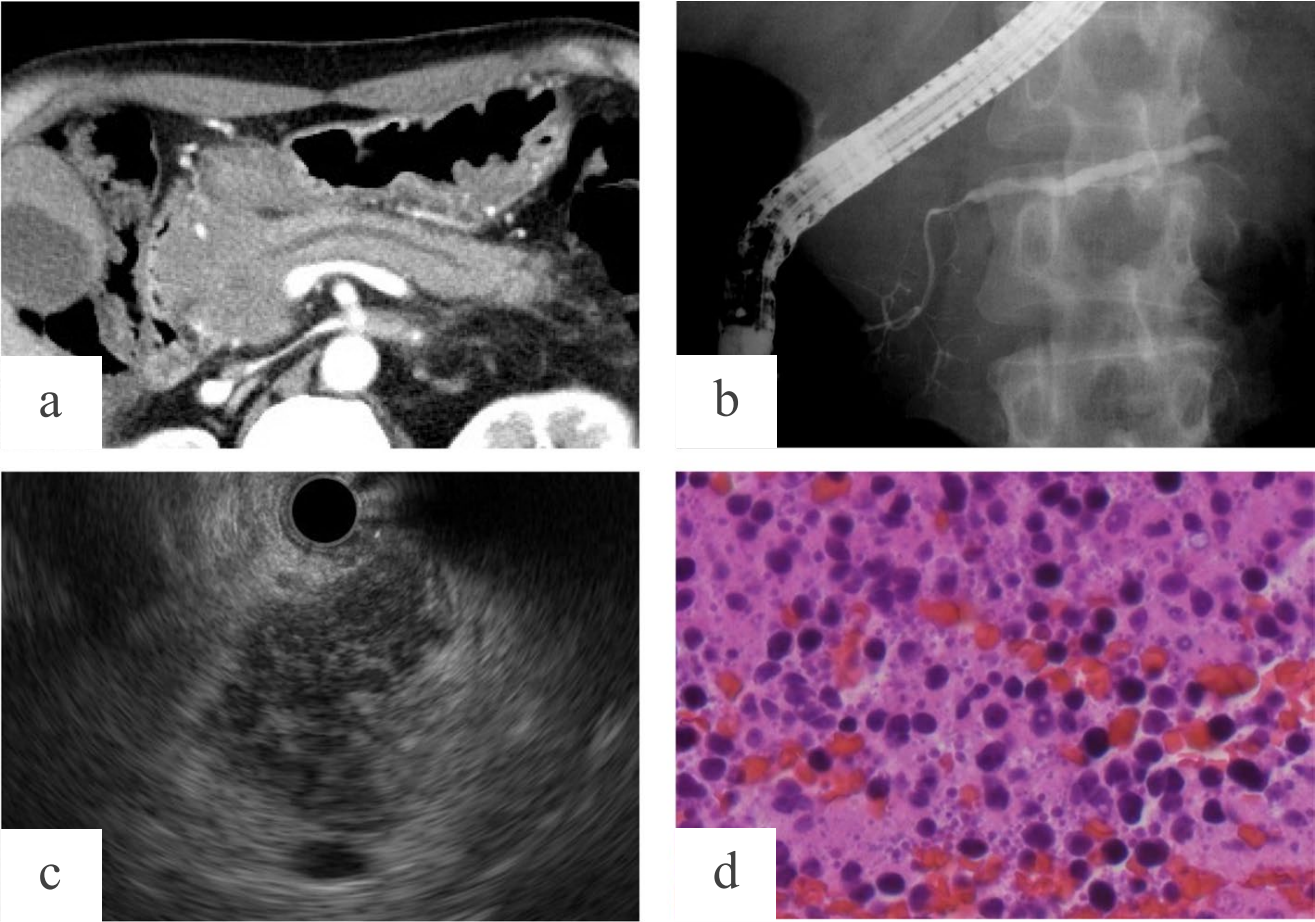
Case 2.1: A case of pancreatic lymphoma that was not definitively diagnosed from specimens obtained by endoscopic ultrasonography–guided fine-needle aspiration. A 65-year-old man was admitted to our hospital with acute pancreatitis. Contrast-enhanced computed tomography showed a homogeneous mass in the pancreatic head measuring 55 mm in maximum diameter and slight dilation of the caudal main pancreatic duct (MPD) at 3.42 mm (**a**). In endoscopic retrograde pancreatography, the MPD localized in the tumor was visible and narrowing smoothly, and the caudal MPD was mildly dilated (**b**). Endoscopic ultrasonography found a 50 mm round hypoechoic tumor with a clear boundary and scattered high echoic spots (**c**) suggesting autoimmune pancreatitis. Cytologic assessment of the tumor using EUS-FNA revealed atypical lymphocyte accumulation suggesting malignant lymphoma (**d**)

**Fig. 4:**
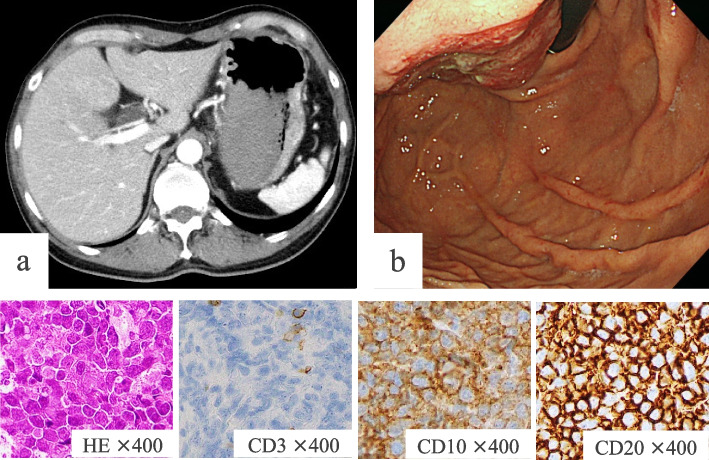
Case 2.2: Contrast-enhanced computed tomography (**a**) and esophagogastroduodenoscopy (**b**) revealed a nodular mass on the gastric fundus wall. The pathological and immunohistochemical examination results of biopsy samples were as follows: CD10 ( +), CD20 ( +), CD79a ( +), CD3 ( −), CD5 ( −), Cyclin D1 ( −), and BCL-2 ( −). The patient was then diagnosed as diffuse large B-cell lymphoma–like Burkitt’s lymphoma

Non-Hodgkin’s lymphomas originating from extralymphatic organs may involve the pancreas, with a frequency of 30% [1]. Most pancreatic involvements arise at an advanced stage, and few cases are found at initial diagnosis. Although pathological examination by endoscopic ultrasonography–guided fine-needle aspiration (EUS-FNA) has been reported to be useful in diagnosing pancreatic lymphoma, some cases can hardly be differentiated from pancreatic ductal adenocarcinoma (PDAC) [2, 3]. We reported a case of malignant lymphoma (ML) prediagnosed with PDAC by EUS-FNA (case 1) [4] (Figs. 1 and 2) and another case that was not definitively diagnosed with ML because only atypical lymphocytes suspicious of ML were obtained from EUS-FNA (case 2) (Figs. 3 and 4). Therefore, not only pathological examination but also clinical and radiological features should be determined when diagnosing ML with pancreatic involvement. Thus, we reported a case of diffuse large B-cell lymphoma involving the pancreatic head, which was discovered through the onset of acute pancreatitis, at initial diagnosis. Further, we conducted a literature review focusing on clinical features and imaging examinations. We also conducted a study using the data of our patients with ML and resected PDAC located in the pancreatic head to analyze differences in clinical features and image examinations between these two malignancies, concentrating on tumor size and caudal main pancreatic duct (MPD) diameter, and serum levels of carbohydrate antigen 19–9 (CA19-9) and soluble interleukin-2 receptor (sIL-2R).

## Methods

### Participants

This retrospective study included 8 patients diagnosed with ML that had a pancreatic head lesion at initial diagnosis and 46 patients with resected PDAC in the pancreatic head between April 2006 and October 2021 at the National Hospital Organization Kure Medical Center and Chugoku Cancer Center. This study conformed to the principles of the Declaration of Helsinki, with approval from our ethics committee (No.2021–64). Patients who had a tumor located at the pancreatic body, tail, groove area, or uncinate process or a tumor that had spread widely beyond the area of the pancreatic head were excluded. We retrospectively reviewed the collected data. ML was diagnosed by a lymph node biopsy, extranodal lesion biopsy, or bone-marrow aspiration and biopsy, whereas PDAC was pathologically diagnosed by a microscopic examination of tissues collected at pancreaticoduodenectomy. Patients’ informed consent for study participation was not required because we used anonymous clinical data that were obtained after each of them agreed for pancreatic cyst surveillance. For disclosure, the details of our study are posted in public areas at the National Hospital Organization Kure Medical Center and Chugoku Cancer Center.

### Tumor size and MPD diameter

Tumor size and MPD diameter were measured at the maximum length based on the axial computed tomography (CT) image. Maximal MPD diameter was measured in the pancreatic body or tail; a diameter over 3 mm indicated MPD dilatation. The association between tumor size and caudal MPD diameter was investigated through the analysis of covariance.

### Serum levels of sIL-2R and CA19-9 in ML and PDAC

We measured the serum levels of sIL-2R in 8 and 46 patients, and CA19-9 in 5 and 46 patients with ML and PDAC, respectively. These data were used to analyze the ability of these biomarkers to differentiate ML from PDAC.

### Receiver operating characteristic (ROC) curve analysis

The ability of sIL-2R and CA19-9 to predict the presence of ML or PDAC was evaluated by ROC curve analysis.

### Statistical analysis

We used Fisher’s exact test and χ^2^ test to analyze the categorical variables, and the Welch *t*-test and Mann–Whitney *U*-test for the quantitative data, where appropriate. To evaluate the usefulness of sIL-2R and CA19-9 for predicting ML or PDAC, we plotted ROC curves and calculated the areas under the curve (AUCs) with 95% confidence interval (CI). Then, we statistically evaluated the AUC and analyzed the differences between biomarkers using the χ^2^ test. Youden’s index was defined for all points of the ROC curve, and its maximum value was used as a criterion for selecting the optimum cutoff point.

All statistical data were analyzed using the Excel statistical software package (Ekuseru-Toukei 2015 version; Social Survey Research Information Co., Ltd., Tokyo, Japan).

## Results

### Patient characteristics

Table 1 shows the characteristics of 54 patients who were diagnosed with ML (*n* = 8) and PDAC (*n* = 46). Among the patients with ML, 38% were male, and the median age was 77 (59–82) years. In those with PDAC, 48% were male, and the median age was 70 (42–84) years. In univariate analysis, the serum levels of alanine aminotransferase (*P* = 0.0259), total bilirubin (*P* = 0.0133), hemoglobin A1c (*P* = 0.0146), and MPD diameter (*P* < 0.001) were higher in PDAC than in ML. In contrast, those with ML had a higher serum sIL-2R level (*P* < 0.001) and a larger tumor size (*P* < 0.001) than those with PDAC.

**Table 1:** Patients’ characteristics in patients with pancreatic ductal adenocarcinoma and malignant lymphoma

	PDAC	ML	*P* value
Patient’s number	46	8	
Age, years, median (range)	70 (42–84)	77 (59–82)	
Male sex, n (%)	22 (48)	3 (38)	0.99
Symptoms			
Upper abdominal pain, n (%)	12 (26)	2 (25)	1.00
Acute pancreatitis, n (%)	3 (6)	2 (25)	0.15
Obstructive jaundice, n (%)	24 (52)	0 (0)	0.0063
Comorbidities			
Diabetes, n (%)	15 (33)	0 (0)	0.09
Hypertension, n (%)	16 (35)	2 (25)	0.70
Dyslipidemia, n (%)	11 (24)	2 (25)	1.00
Family history of pancreatic cancer (≦2^nd^ degree), n/N (%)	4/36 (11)	0/4 (0)	1.00
Smoking, n/N (%)	16/44 (36)	2/7 (29)	1.00
Alcohol drinking, n/N (%)	22/45 (49)	3/7 (43)	1.00
Laboratory data			
Aspartate aminotransferase (U/L)	138 ± 147	53 ± 61	0.16
Alanine aminotransferase (U/L)	201 ± 222	68 ± 130	0.026
Lactate dehydrogenase (U/L)	259 ± 77	406 ± 330	0.12
Alkaline phosphatase (U/L)	1046 ± 1075	563 ± 589	0.093
γ-glutamyl transferase (U/L)	547 ± 679	192 ± 589	0.088
Total bilirubin (mg/dL)	4.87 ± 5.43	0.93 ± 0.70	0.013
Amylase (IU/L), (patient’s number)	99 ± 87	436 ± 790 (6)	0.48
White blood cell count (× 10^2^/μL)	63.0 ± 19.8	71.3 ± 40.1	0.91
C-reactive protein (mg/dL)	0.78 ± 1.00	2.06 ± 3.06	0.98
HbA1c (%)	6.6 ± 1.6 (*n* = 45)	5.3 ± 0.4 (*n* = 6)	0.015
sIL-2R (U/mL)	467 ± 143	1321 ± 596	< 0.001
CA19-9 (U/mL)	779 ± 1628 (*n* = 46)	42 ± 47.4 (*n* = 5)	0.086
CEA (ng/mL)	5.88 ± 6.36	2.44 ± 1.22 (*n* = 7)	0.058
Tumor size, mm, mean ± SD	24.92 ± 8.11	45.88 ± 12.33	< 0.001
Diameter of main pancreatic duct, mm, mean ± SD	6.54 ± 2.56	2.47 ± 0.79	< 0.001

Laboratory data was shown as mean ± SD. We had several data defectiveness and ‘n’ in table show analyzed patient’ number and ‘n/N ‘in table show positive number/analyzed number

*sIL-2R* soluble interleukin-2 receptor, *CA19-9* Carbohydrate antigen 19–9, *CEA* carcinoembryonic antigen, *PDAC* pancreatic ductal adenocarcinoma, *ML* malignant lymphoma

### Tumor size and MPD diameter

**Fig. 5:**
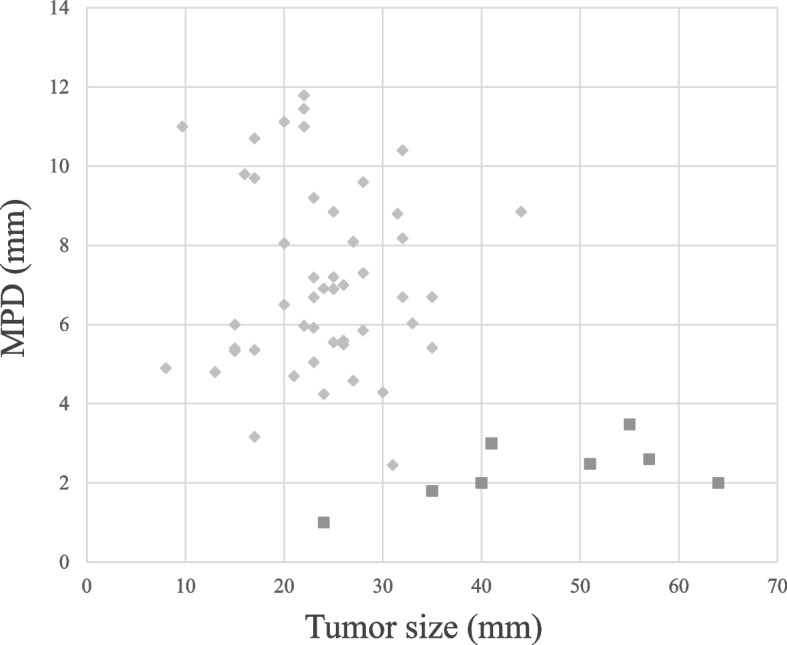
Tumor size and the diameter of the caudal main pancreatic duct (MPD) on computed tomography according to the analysis of covariance. The MPD diameter relevant to tumor size was significantly smaller in the malignant lymphoma (*n* = 8) than in the pancreatic ductal adenocarcinoma (*n* = 46)

The median tumor size was 45.8 (24–64) mm in ML while 23.9 (8–44) mm in PDAC, and the median diameter of caudal MPD was 2.5 (1.0–3.5) mm in ML and 7.1 (2.5–11.8) mm in PDAC. Both clinical features showed significant differences between the two malignancies (both, *P* < 0.001). In the analysis of covariance, ML had a smaller caudal MPD per tumor size than PDAC, demonstrating a statistical difference (*P* = 0.042) (Fig. 5).

### Serum levels of sIL-2R and CA19-9 in ML and PDAC

**Fig. 6:**
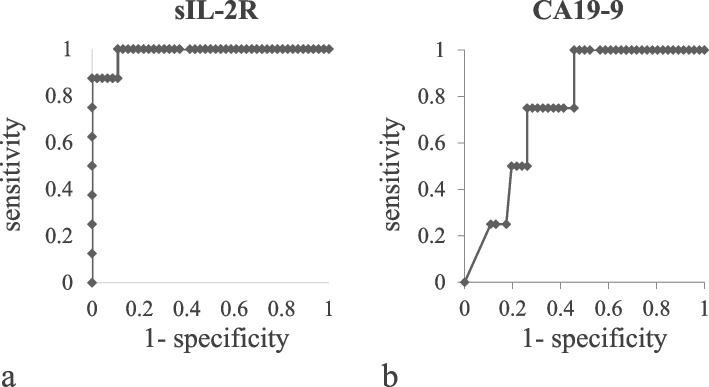
Efficacy of the serum levels of sIL-2R and CA19-9 in differentiating malignant lymphoma (ML) from pancreatic ductal adenocarcinoma (PDAC) according to the receiver operating curve analysis: **a** sIL-2R for differentiating ML (*n* = 8) from PDAC (*n* = 46) (area under the curve [AUC] 0.99, 95% confidence interval [CI] 0.96–1.02, *P* < 0.001); **b** CA19-9 for differentiating PDAC (*n* = 46) from ML (*n* = 5) (AUC 0.76, 95% CI 0.57–0.95, *P* = 0.007)

The median serum levels of sIL-2R were 1321 (658–2235) and 467 (238–804) U/mL in ML and PDAC, and those of CA19-9 were 34 (2–122) and 779 (2–8369) U/mL, respectively. Both features showed significant differences between patients with ML and PDAC (both, *P* < 0.001). In addition, the ROC analysis showed that each of them was useful for differentiating ML from PDAC (sIL-2R: AUC 0.99, 95% CI 0.96–1.02, *P* < 0.001; CA19-9: AUC 0.76, 95% CI 0.57–0.95, *P* = 0.007) (Fig. 6a, b). The optimal cutoff point of sIL-2R was 658 U/mL (100.0% sensitive, 89.1% specific), and that of CA19-9 was 32 U/mL (73.9% sensitive, 66.7% specific). As calculated by the Youden method, the cutoff value of CA19-9 was 37 U/mL, which is generally considered as abnormal, and that of sIL-2R was 658 U/mL. Moreover, the sensitivity and specificity values according to sIL-2R ≥ 658 U/mL plus CA19-9 < 37 U/mL for differentiating ML from PDAC were 80.0% and 95.6%, respectively.

## Discussion

Some cases may be misdiagnosed as PDAC when atypical cells are detected in pancreatic tumor specimens by EUS-FNA. Some studies have collected case reports and reviewed the characteristics or imaging findings of ML in the pancreas [1, 4–7]. However, we found no reports directly comparing the clinical features and images of PDAC cases with those of ML cases from a single institution. Thus, our report might be the first to verify and report the clinical features and imaging characteristics of ML and PDAC cases at our institution.

Generally, 25% of non-Hodgkin’s lymphomas originate from extralymphatic organs, and approximately 30% of them may involve the pancreas [1]. Diffuse large B-cell lymphoma is the most common histological subtype, and the pancreatic head is the most common occupied location [5]. Although cases accompanied with lymphadenitis in the neck, mediastinum, and inguinal region can be easily diagnosed as ML, those with sole pancreatic involvement can be difficult to arrive at pancreatic lymphoma diagnosis. CT is by far the most common imaging technique for detecting and characterizing pancreatic lymphoma. In contrast-enhanced CT images, most lymphomas are shown as well-defined, sometimes bulky and infiltrating, homogeneous low-attenuation masses relative to the pancreatic parenchyma with only mild enhancement [6]. Some ML cases can hardly be differentiated from PDAC, neuroendocrine neoplasm (NEN), solid pseudopapillary neoplasm (SPN), acinar cell carcinoma (ACC), and autoimmune pancreatitis. Given the high frequency of pancreatic diseases, differentiating ML from PDAC is crucial to avoid unnecessary surgical interventions.

Pathological examination by EUS-FNA is reportedly useful for diagnosing pancreatic lymphoma [2]. However, we sometimes have difficulty in arriving at ML diagnosis because obtaining sufficient histologic specimen is challenging. A definite diagnosis of ML needs immunohistochemical examinations. Sometimes, cytological assessment can also difficultly distinguish ML from poorly differentiated PDAC, NEN, and other similar conditions. Moreover, sampling errors can occur from atypical glandular cells of the intestinal tract or normal pancreatic parenchyma in the puncture route of EUS-FNA. In case 2, cytological examination by EUS-FNA showed an accumulation of atypical lymphocytes, which suggest ML; however, this result was insufficient to make a diagnosis of any ML subtype because of the absence of histological assessment.

We usually underwent EUS-FNA or fine-needle biopsy (FNB) for pancreatic mass using a 22- or 25-gauge Menghini-tip needle with a standard method (dry suction), and a rapid on-site evaluation is performed. Sometime, a 22-gauge reverse bevel needle is used for patients with suspected ML, NEN, ACC, and SPN. In most cases, specimens appropriate for histologic evaluation are not obtained, and immunohistological evaluation is performed using cell clot from EUS-FNA. In recent years, several studies have reported progressive improvements in tissue acquisition of pancreatic masses, including the use of needles of different calibers and types, number of needle passes, and different sampling techniques, such as slow-pull method and wet-suction technique [8–11]. A new type of needle (end-cutting needle) and wet-suction technique showed excellent histological yields [11]. EUS elastography can detect pancreatic areas with increased stiffness within a focal lesion to better target the needle during EUS-FNA or FNB [12]. These techniques can potentially improve sample collections.

Indeed, difficulties in the pathological diagnosis of ML continue to exist. Thus, clinical features and image examinations in addition to pathological tests are necessary to accurately diagnose ML.

We searched for cases of ML involving the pancreas by using PubMed and Igakuchuozassi (Japanese) between 2000 and 2021. We used the keywords “pancreas,” “malignant lymphoma,” and “pancreatic lymphoma” in PubMed and “*suizou*,” “*akuseirinnpasyu*,” “*suiakuseirinnpasyu*,” “pancreatic lymphoma,” “pancreas,” and “*akuseirinnpasyu*” in Igakuchukozassi. Twelve cases were then collected. Subsequently, we analyzed the clinical and pathological features of ML localized in the pancreatic head in these 12 cases and our 2 cases. All cases had tumors in the pancreatic head. Six of these cases (42.9%) were diagnosed as the onset of acute pancreatitis. Acute pancreatitis has been reported as an initial presenting symptom of pancreatic cancer [7]. Although there was no significant difference between these malignancies (25% vs. 6%, *P* = 0.15), we presumed that MPD stenosis is milder in ML than in PDAC and that the following residual transitability of pancreatic juice might induce frequent acute pancreatitis in ML. In addition, most MLs are located in the pancreatic head, but obstructive jaundice is an infrequent symptom of ML [6]. As shown in Table 1, our patients with ML showed significantly lower levels of total bilirubin than PDAC (*P* = 0.01). Mild stenosis of the common bile duct might also induce a lower frequency of jaundice.

Merkle et al. reported some useful features on CT to differentiate ML from PDAC [6]: (1) a localized pancreatic head tumor without significant MPD dilatation, (2) invasive and infiltrating tumor growth through the retroperitoneal or upper abdominal organs and the gastrointestinal tract, and (3) presence of enlarged lymph nodes below the level of the renal veins. Furthermore, magnetic resonance imaging (MRI) and positron emission tomography (PET)–CT findings may be helpful for the differential diagnosis of lymphoma involving the pancreas from other lesions including PDAC [13, 14]. In MRI, lymphoma shows a significantly restricted diffusion similar to the spleen. In PET-CT, fluorodeoxyglucose is more substantially accumulated in pancreatic lymphoma than in inflammatory diseases such as autoimmune pancreatitis and other neoplasms, including PDAC and neuroendocrine tumors [13]. Further, several studies have reported the enhanced pattern of ML in enhanced-contrast EUS but there is no decided pattern [15]. This unfortunate result is thought that ML has various pathological type [15]. Of the 14 cases we reviewed, 8 had no MPD dilatation, while 6 showed mild dilatation. Moreover, all cases registered in our hospital with lymphoma of the pancreatic head showed no significant MPD dilatation, as shown in Fig. 5. Table 1 shows that none or mild MPD dilatation is a useful characteristic in differentiating ML from PDAC.

When a mass is encountered in the pancreatic head without jaundice or pancreatic ductal dilatation, lymphoma should be considered. Involvement of clinical findings (absence of jaundice) and tumor markers, specifically elevated sIL-2R level and normal CA 19–9 level, further strengthens the likelihood of lymphoma over PDAC, warranting diagnostic biopsy with requisite histologic and immunophenotypic examinations. In ML diagnosis, higher sIL-2R levels strongly suggest the presence of a lymphoma, thereby possibly useful for the diagnosis of lymphomas, but other false-positive conditions should be ruled out first [16]. Furthermore, the serum biomarker CA19-9 is currently the gold standard for PDAC diagnosis [17]. Pancreatic cancers sometimes show high sIL-2R levels because this biomarker is elevated in several conditions including inflammation. Therefore, sIL-2R levels might appear to be a less specific marker for ML, although adding more ML-related factors improves the diagnostic accuracy of ML. In our study, the combination of sIL-2R and CA19-9 levels was highly sensitive for ML and PDAC differentiation. The PDAC group had fewer patients with high sIL-2R levels but had more patients with very high CA19-9 levels than the ML group. In comparison, most patients with ML showed normal serum levels of CA19-9. These features might be useful when differentiating ML from PDAC.

## Conclusions

In conclusion, diagnosing pancreatic ML can be difficult in some patients, even by EUS-FNA. Thus, clinical and imaging features should also be determined. ML may be considered if the patient has pancreatic lesions with acute pancreatitis, no obstructive jaundice, no or slight caudal MPD dilatation, and high sIL-2R level plus low CA19-9 level. However, in the absence of a definite diagnosis of adenocarcinoma, such as atypical glandular cells, by EUS-FNA, re-examination may be prudent.

## Data Availability

The datasets supporting the conclusions of this article available from the corresponding author, NK, on reasonable request.

## Abbreviations

AUC: Areas under the curve
CI: Confidence interval
CT: Computed tomography
ML: Malignant lymphoma
MPD: Main pancreatic duct
MRI: Magnetic resonance imaging
PET: Positron emission tomography
ROC: Receiver operating characteristic
